# Higher diet quality is associated with a lower prevalence of MASLD and adverse health outcomes: insights from NHANES 2005 to 2020

**DOI:** 10.1007/s00394-025-03809-4

**Published:** 2025-10-06

**Authors:** Ayesha Sualeheen, Sze-Yen Tan, Robin M. Daly, Ekavi Georgousopoulou, Stuart K. Roberts, Elena-S. George

**Affiliations:** 1 Institute for Physical Activity and Nutrition (IPAN), School of Exercise and Nutrition Sciences, Deakin University, Geelong, 3220 Australia; 2 School of Medicine Sydney, University of Notre Dame Australia, Sydney, NSW Australia; 3 Discipline of Nutrition and Dietetics, Faculty of Health, University of Canberra, Canberra, Australia; 4 Department of Gastroenterology, Alfred Health, Melbourne, VIC Australia; 5 School of Translational Medicine, Monash University, Melbourne, VIC Australia

**Keywords:** Diet quality, MASLD, HEI-2020, DASH, MedDietScore, Mortality

## Abstract

**Purpose:**

MASLD affects over 30% of the global population increasing the risk of adverse health outcomes. Improvements in diet quality (DQ) may offer a modifiable solution. This study explored the association between DQ and MASLD prevalence, advanced fibrosis, and all-cause mortality.

**Methods:**

Participants from the NHANES U.S. national database were included in the cross-sectional (n = 20,502) and longitudinal (n = 13,161) analysis. DQ assessed using Healthy Eating Index (HEI)-2020, Mediterranean Diet Score (MedDietScore), and Dietary Approach to Stop Hypertension (DASH). MASLD and advanced fibrosis identified by the Fatty Liver Index and NAFLD fibrosis score, respectively. Mortality was ascertained by the National Death Index. Multivariate logistic regression and Cox proportional hazards analysis estimated odds ratios (OR) for MASLD and hazard ratios (HR) for mortality, with 95% confidence interval.

**Results:**

Each 1-SD increase (improvement) in HEI-2020 and MedDietScore was associated with 25% lower likelihood of MASLD, while DASH showed 31% lower likelihood. Each 1‑SD increase in HEI‑2020, MedDietScore, and DASH was associated with lower ORs for advanced fibrosis in the MASLD group: 0.83 (0.74–0.94), 0.84 (0.75–0.95), and 0.78 (0.68–0.89), respectively. Over 9.3 years’ median follow‑up, higher DQ was associated with lower all‑cause mortality in the overall population [HEI‑2020: 0.93 (0.86–0.99); MedDietScore: 0.92 (0.86–0.99); DASH: 0.89 (0.83–0.96)], with similar trends in the non‑MASLD group but not in those with MASLD or advanced fibrosis.

**Conclusions:**

Higher DQ was associated with lower likelihood of MASLD and advanced fibrosis in U.S. adults and was protective against all-cause mortality in overall and non-MASLD population but not in those with MASLD.

**Supplementary Information:**

The online version contains supplementary material available at 10.1007/s00394-025-03809-4.

## Introduction

Over the past 40 years, metabolic dysfunction-associated steatotic liver disease (MASLD) has become the most widespread chronic liver disorder. MASLD affects over 30% of the global population, and by 2040, its prevalence is forecasted to increase to 55% [1]. MASLD is defined by steatosis affecting > 5% of the hepatocytes, accompanied by one or more cardiometabolic risk factors [2]. Obesity and insulin resistance, mainly stemming from an imbalance between energy intake and expenditure fuels the pathophysiological mechanism driving MASLD onset and progression [3]. Although MASLD has increased in tandem with the obesity epidemic, it can also affect people with normal body weight who have other metabolic abnormalities [4] Notably, those with insulin resistance and steatosis have a two-fold higher risk of developing adverse hepatic outcomes, including advanced fibrosis, cirrhosis, and mortality [5]. A 2025 meta-analysis found that the highest rates of MASLD-related cirrhosis were reported in North America and Australia, followed by Europe and Asia [6]. In the United States, MASLD-related hepatocellular carcinoma (HCC) deaths are rising rapidly and projected to become the second leading cause of HCC-related deaths by 2032 [7]. People with MASLD are dying prematurely, which could be significantly reduced through targeted and sustainable lifestyle modifications.

Lifestyle modification currently remains the mainstay of MASLD management, and while Mediterranean (Med) diet may be the preferred choice, specific dietary composition has not yet been standardised and differences in dietary recommendations persist across clinical practice guidelines [8]. Poor or low-quality diet characterised by excessive intake of ultra-processed foods, red meat, refined grains, unhealthy fats, and sugar sweetened beverages increases the likelihood of MASLD by up to 50% [9]. Observational studies suggest that consumption of ultra‑processed foods and foods with added fructose is associated with 42% [10] and 31% [11] higher odds for MASLD, respectively. However, controlled feeding trials indicate that fructose has no adverse effect on hepatic steatosis or transaminase levels when consumed within isocaloric diets, but may lead to these adverse outcomes within the context of hypercaloric diets [12]. Such dietary patterns may contribute to MASLD pathogenesis by increasing flux of free fatty acids, stimulating de novo lipogenesis, blocking hepatic β oxidation, inducing cellular stress, intestinal dysbiosis and activating pro-inflammatory pathways leading to constant liver injury [13–15].

The Healthy Eating Index (HEI), the Mediterranean Diet Score (MedDietScore), and the Dietary Approaches to Stop Hypertension (DASH) score are diet quality (DQ) scores, developed from established dietary guidelines or patterns, emphasising food groups associated with health benefits [16–18]. Similarities across all three scores include the emphasis on high intakes of fruits, vegetables, whole grains, and plant- and seafood-based protein sources, alongside limiting refined carbohydrates, sodium, and saturated fats [16–18]. Key differences pertain to the inclusion of alcohol, the differentiation of dairy products based on fat content, and variations in the types and quantity of fats and protein sources [16–18]. The HEI‑2020 is the latest iteration of the HEI, aligned with the Dietary Guidelines for Americans (DGA) 2020–2025, assesses DQ independent of quantity [16]. Higher HEI scores have been associated with a lower likelihood of steatotic liver diseases, with metabolic dysregulations significantly mediating this relationship [19, 20]. The MedDietScore assesses adherence to the traditional Med diet, which has been associated with hepatic and cardioprotective benefits, independent of weight loss, in the MASLD population [17, 21, 22]. These benefits extend beyond the Mediterranean region, where the pattern has not been habitually consumed [23] and associated with reduced rates of cardiovascular disease, a leading cause of death in MASLD [24, 25]. The DASH score evaluates adherence to the DASH dietary pattern, which is promoted by the US National Heart, Lung, and Blood Institute as a lifestyle approach to prevent and manage hypertension [18, 26]. Meta-analyses suggests that the DASH diet is associated with significant improvements in cardiometabolic risk factors including in individuals with MASLD [27, 28] suggesting its potential to mitigate adverse health outcomes in this population.

Clinical guidelines recommend 7–10% of weight loss, achieved through a 500–1000 cal deficit from diet and physical activity, for histopathological improvement in MASLD [29–31]. Therefore, diets primarily targeting weight loss such as low-fat, low carbohydrate, ketogenic and/or very low carbohydrate diets have been utilised for MASLD treatment. However, these diets, often high in animal protein or fat, may not be cardioprotective, require strict medical supervision, and lack strong evidence for long‑term benefits or safety [32]. In contrast, dietary patterns such as the HEI, Med diet, and DASH provide a flexible, balanced, and evidence-based approach that can be tailored to individual needs and preferences [27, 33, 34]. Despite emerging interest, there is limited evidence on the effectiveness of these dietary patters in improving MASLD outcomes, including advanced fibrosis and mortality. Therefore, this study aims to assess the association of HEI-2020, MedDietScore and DASH with MASLD prevalence and advanced fibrosis in the National Health and Nutritional Examination Survey (NHANES) of the United States (U.S.) national database (2005–2020). A secondary aim is to assess the association of these DQ tools with all-cause mortality in those with and without MASLD and advanced fibrosis.

## Methods

This study utilised data from NHANES conducted by the Centre for Disease Control and Prevention and the National Centre for Health Statistics (NCHS) of the U.S. The NHANES survey employs complex, multistage probability sampling design to provide nationally representative estimates on the health and nutritional status of noninstitutionalized civilian population of U.S. Briefly, NHANES includes data on demographic, health examination, laboratory and dietary assessments for participants of all ages. The methodology and data collection protocols and data files of NHANES are publicly available elsewhere [35].

**Fig. 1:**
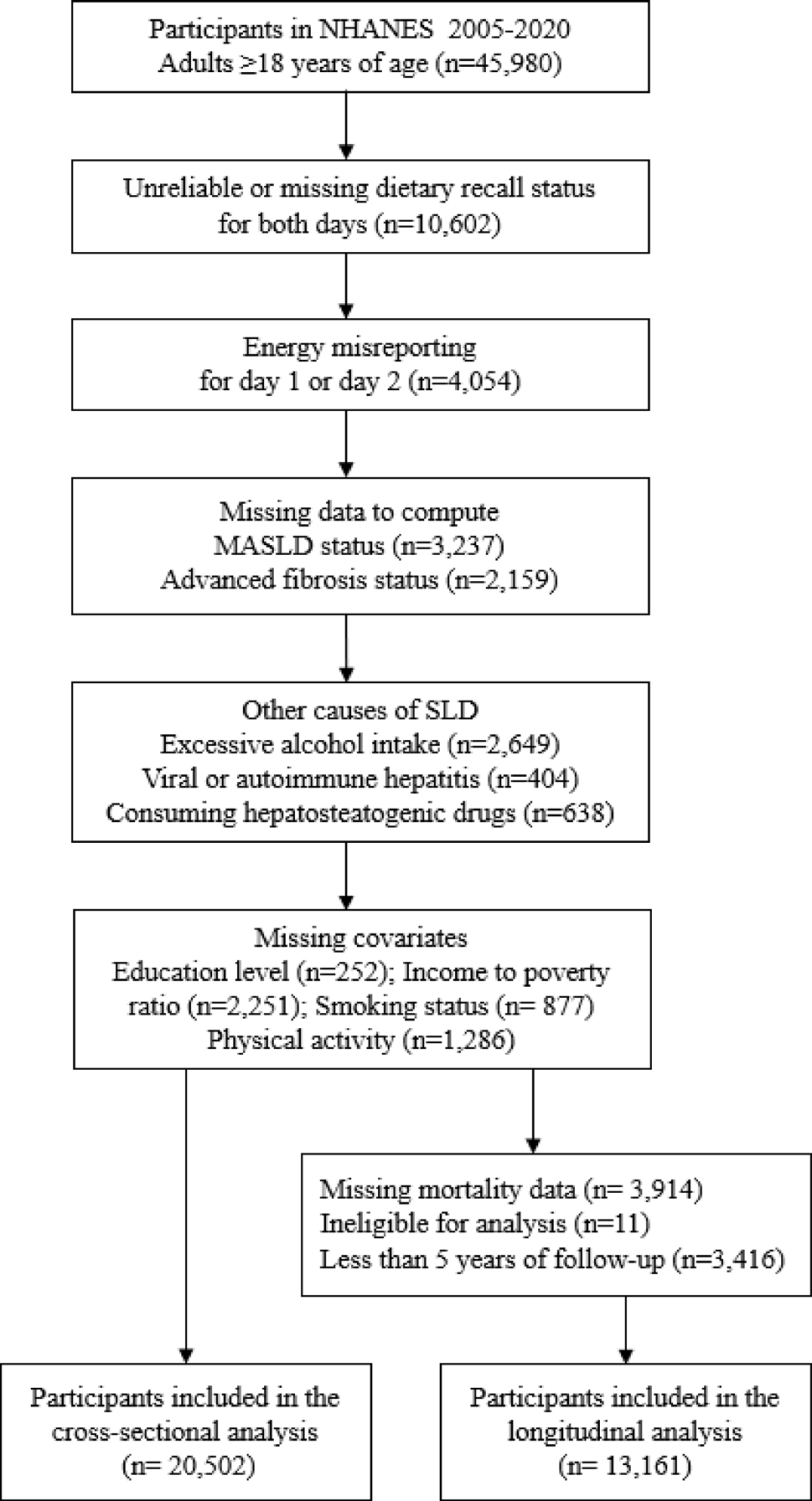
Study flow chart for participants inclusion from NHANES for cross-sectional and longitudinal analysis. MASLD, metabolic dysfunction associated fatty liver disease; NHANES, national health and nutritional examination survey; SLD, steatotic liver disease

For the current study, we included participants from NHANES 2005 to 2020 cycles aged ≥ 18 years and who had two reliable 24‑h dietary recalls available, with inclusion and exclusion criteria illustrated in Fig. 1. Those with unreliable or missing dietary recall(s) and with implausible dietary kilocalories (kcal) defined as outside the range of 500–3500 kcal/day for females and 800–4200 kcal/day for males were excluded [36]. The following were also excluded: participants with missing data required to compute MASLD status, presence of other causes of hepatic steatosis including excessive alcohol consumption (> 30 g/day for men; > 20 g/day for women), use of hepatosteatotic drugs, and presence of viral or autoimmune hepatitis. Participants with missing covariate information were also excluded. In total, 20,502 participants with complete data met the inclusion criteria for the cross-sectional analysis (Fig. 1).

For the longitudinal analysis, the public access linked mortality files from NCHS were merged with the demographic files and, mortality data was used from NHANES 2005 until the 2016 cycle. NHANES assigned different respondent sequence identification numbers (SEQNs) for 2017–2020 to prevent the separation of 2017–2018 data from 2019–2020 cycle, as the latter was not nationally representative. Consequently, the 2017–2018 mortality data (which matches the SEQNs for the 2017–2018 demographic files) could not be merged in the present study [19]. For the longitudinal analysis, participants with missing mortality data, insufficient identifying information for data linkage, or less than five years of follow-up were excluded. In total, 13,112 participants were included for the longitudinal analysis (Fig. 1).

The NHANES study was approved by the National Centre for Health Statistics review board, and all participants provided written informed consent.

### Dietary intake assessment

Dietary intake in NHANES assessed through two 24‑h dietary recalls, collected over two non‑consecutive days (including both weekdays and weekends) using the U.S. Department of Agriculture’s (USDA) Automated Multiple‑Pass Method [37]. The first recall is conducted in person at the Mobile Examination Centre, and the second by telephone 3–10 days later. A detailed description of the NHANES dietary data collection protocol has been provided elsewhere [37].

In this study, the USDA’s Food Pattern Equivalent Database (FPED), which translate the foods and beverages reported in each 24-h recall of NHANES participants into 37 food pattern components was used to compute DQ [38]. The food patterns were quantified as cup equivalents for fruits, vegetables, and dairy; ounce equivalents for grains and protein foods; teaspoon equivalents for added sugars; gram equivalents for solid fats and oils; and the number of alcoholic beverages consumed [38]. Single foods were assigned directly into their respective food pattern components whereas, combination foods (multi-ingredient items) were disaggregated into single ingredients and assigned to an appropriate food pattern component [38]. The FPED data have been utilised in prior studies to assess DQ of participants across various NHANES cycles [39, 40].

The FPED files for two 24-h dietary recalls for each NHANES cycle (2005–2020) were extracted, which were then merged with the demographic and nutrient intake data of participants via unique identifier. The food patterns were then standardised into respective serving equivalents based on the criteria defined by the DQ indices used in this study, and the average of both days was used for DQ analysis [40].

### Diet quality assessment

The DQ indices employed in this study were the HEI-2020 [16] MedDietScore [17] and DASH [18]. The HEI-2020 consists of 13 adequacy and moderation components which includes, total fruits, whole fruits, total vegetables, greens and beans, whole grains, dairy, total protein foods, seafood and plant proteins, fatty acids, refined grains, added sugars, saturated fats, sodium[16]. The total HEI-2020 score ranged between 0 and 100, where higher scores indicative of greater adherence towards DGA 2020 recommendations [16]. The MedDietScore is designed to assess weekly consumption of foods that closely align with a Med dietary pattern, such as high intakes from non-refined cereals, fruits, vegetables, legumes, olive oil, fish and potatoes and foods to limit, including meat products, poultry and full fat dairy, and alcohol in moderation[17]. The total MedDietScore ranges between 0 and 55, where higher scores are indicative of an increased adherence towards Med dietary pattern [17]. The DASH score consists of eight dietary components, including foods to encourage, such as fruits, vegetables (excluding potatoes and legumes), whole grains, nuts, legumes, and low-fat dairy, and foods to limit, such as red and processed meats, sweetened beverages, and sodium[18]. The total score for DASH ranged between 8 and 40, where higher scores are reflective of better adherence to the DASH dietary pattern [18]. These DQ indices offer a validated and practical assessment of overall dietary patterns and their association with health outcomes [41]. They summarise multiple components into a crude score and are adaptable to various dietary assessment methods. However, they may be population-specific, require specific intake data, and only measure adherence to selected components, overlooking the synergistic structure of food matrix [41].

Some dietary components in these DQ indices were not directly available in the FPED dataset (e.g., low-fat dairy and sugar-sweetened beverages for DASH; olive oil and its consumption frequency for MedDietScore). In such cases, corresponding USDA food codes were identified using the Food and Nutrient Database for Dietary Studies and the What We Eat in America Food Categories (NHANES) [42], and then grouped into their respective components, with gram weights from 24-h dietary recalls converted into serving equivalents [40]. Due to the absence of data on olive oil consumption frequency in the NHANES dietary dataset, this component of the MedDietScore was substituted with the ratio of monounsaturated to saturated fatty acids (MUFA: SFA), in combination with olive oil intake expressed in grams. An optimal cutoff of a MUFA:SFA ratio of 2.0, reflecting approximately 20% of total energy intake from MUFAs and ≤ 10% from SFAs, was applied. [43, 44]. The scoring criteria for each DQ index used in this study has been provided in the supplementary file (**S1**).

### MASLD and advanced fibrosis

The presence of hepatic steatosis was confirmed using the non-invasive Fatty Liver Index (FLI) test that is calculated based on body mass index (BMI), waist circumference and serum triglyceride and gamma-glutamyl transferase levels [45]. MASLD was identified in individuals with hepatic steatosis (FLI ≥ 60) and at least one of five cardiometabolic risk factors, as defined by expert panel consensus: overweight or obesity, insulin resistance, hypertension, hypertriglyceridemia and/or low high-density lipoprotein (HDL) levels [2]. Advanced fibrosis was identified using the NAFLD fibrosis score, with a validated cutoff of > 0.676 used to rule-in advanced fibrosis [46].

### Covariate assessment

Sociodemographic covariates included age, sex (males and females), race/ethnicity (non-Hispanic whites, non-Hispanic black, Hispanics, Asians and other race), education level (high school or below and more than high school) and family poverty to income ratio. Type 2 diabetes (T2DM) status was determined through self-reported diagnoses, supplemented by fasting HbA1C levels ≥ 6.5% or the use of antidiabetic drugs [33]. CVD was assessed by self-reported diagnosis of angina/angina pectoris, coronary heart disease, stroke, congestive heart failure, or heart attack.

Lifestyle covariates included smoking and physical activity status. Smoking status was assessed using the SMQ data files with participants classified as current, former or non-smokers, as detailed elsewhere [39]. Self-reported leisure time physical activity data were collected for previous months or weeks across different NHANES cycles, using various questionnaires [47, 48]. To standardise physical activity measures across the eight NHANES cycles (2005–2020), total minutes of leisure-time moderate and vigorous activity were calculated by multiplying activity duration (minutes) by the number of days performed, summing these to obtain daily total physical activity minutes, and converting them into z-scores for each cycle.

### Mortality outcome

The NCHS has linked NHANES cycles (1999–2018) with mortality data from the National Death Index for all participants aged ≥ 18 years [49]. For this study, the public-access linked mortality files, which provide follow-up data from the date of survey participation to the 31 December 2019, were used for NHANES 2005–2016 cycles. Mortality due to any cause was the outcome in this study.

### Statistical analysis

Complete case analysis was employed as the primary method for handling missing data in the statistical analysis. Characteristics of study population presented as weighted means (95% CI) for continuous variables and as proportions for categorical variables. Appropriate survey weights recommended by NHANES were applied to ensure estimates representative of the U.S. civilian non-institutionalized population [50]. NHANES medical examination and dietary weights were adjusted accordingly to account for the combination of multiple NHANES cycles [50]. Independent t-tests were used for comparisons between MASLD and non-MASLD group for continuous variables and Chi-square for categorical variables. Binary logistic regression analysis was used to determine the association between total DQ score, MASLD, and advanced fibrosis in individuals with MASLD. The multivariate model adjusted for age, sex, race, education level, income ratio, smoking status, physical activity, mean energy intake, T2DM and CVD status. Due to variations in total and individual component scores across DQ indices, these scores were standardized to a mean of 0 and a standard deviation of 1 to facilitate comparisons between each index. *Bonferroni* correction was applied to adjust for multiple comparisons across individual DQ components. Statistical significance thresholds were set at *p* < 0.004 for HEI-2020, *p* < 0.005 for MedDietScore, and *p* < 0.006 for DASH, according to the number of individual dietary components in each index. The total scores were further categorized into quartiles, with Q1 (lowest quartile) serving as the reference group.

For longitudinal analysis, Cox proportional hazard analysis with time-in-study as the timescale was used to assess associations between DQ indices and all-cause mortality. Follow-up time was calculated from the baseline interview date to the date of death or the censor date (31st December 2019), whichever came first. To mitigate the risk of reverse causality, the initial 5 years of follow-up were excluded. A subgroup analysis was conducted between individuals with and without MASLD, as well as those with and without advanced fibrosis within the MASLD group using group-specific quartiles for each DQ index. The analysis adjusted for age, sex, race, education level, income ratio, smoking status, physical activity, mean energy intake, T2DM and CVD status. The proportional hazards assumption for the covariates was assessed using Schoenfeld residual plots and no violation of this assumption found in the analysis. BMI was not included as a covariate in either regression analysis to avoid over-adjustment and collider bias [51], as it forms part of the FLI equation and is one of the cardiometabolic components used for MASLD diagnosis.

All analyses were performed by using STATA version 18.0 (StataCorp, College Station, TX, USA), with a *p*-value of < 0.05 was considered statistically significant, unless otherwise stated.

## Results

### Cross-sectional analysis

A total of 20,502 participants were included in the cross-sectional analysis with mean age of 47.7 years and 45.5% males. The weighted MASLD prevalence in NHANES 2005–2020, as assessed by FLI, was 47.8%. In the overall cohort, the prevalence of hypertension, T2DM, and CVD was 35.4%, 12.6%, and 8.7%, respectively (Table 1). Individuals with MASLD had a significantly higher prevalence of hypertension (45.4% *vs* 24.7%), T2DM (20.7% *vs* 5.4%) and CVD (11.9% *vs* 5.9%), as well as lower HDL-C levels and elevated liver enzyme concentrations, compared to those without MASLD (all *p*-values < 0.001). The prevalence of advanced fibrosis was 8.7% in the MASLD group. Additionally, individuals with MASLD had significantly lower HEI-2020 score (51.0 *vs* 54.0), MedDietScore (21.5 *vs* 22.9), and DASH score (21.4 *vs* 23.2) compared to non-MASLD group (Table 1).

**Table 1:** Weighted demographic characteristics of NHANES participants (2005–2020)

Variables	Overall (n = 20,502)	MASLD (n = 9841)	Non-MASLD (n = 10,661)	*P* value*
*Mean (95% CI) unless otherwise reported*				
Age (years)	47.7 (47.2–48.3)	50.3 (49.8–50.9)	45.4 (44.8–46.1)	** < 0.001**
Gender, males, %	45.5	53.3	46.7	
*Race/Ethnicity, %*				
*Non-Hispanic Whites*	67.6	67.4	67.1	** < 0.001**
*Hispanics*	14.2	16.2	12.9	
*Non-Hispanics Blacks*	10.1	10.3	10.0	
*Asians*	4.9	2.6	7.1	
*Others*	3.2	3.6	2.9	
Education level, %				
*Less than or equal to high school*	36.6	40.5	33.1	** < 0.001**
*More than high school*	63.4	59.5	66.9	
Income to poverty ratio	3.1 (3.0–3.2)	3.0 (2.9–3.1)	3.2 (3.1–3.2)	** < 0.001**
BMI (kg/m^2^)	29.3 (29.1–29.4)	34.3 (34.1–34.5)	24.8 (24.7–24.9)	** < 0.001**
*BMI categories, %*				
Under/Normal weight*	27.4	1.1	50.5	** < 0.001**
*Overweight*	32.7	22.1	42.1	
*Obese*	39.9	76.8	7.4	
*Waist circumference (cm)*				
*Males*	102.5 (101–103)	112.6 (112.0–113.2)	91.0 (90.6–91.4)	** < 0.001**
*Females*	97.3 (96.8–97.8)	112.7 (112.2–113.2)	86.5 (86.1–86.9)	** < 0.001**
Hypertension, %	35.4	45.4	24.7	** < 0.001**
Diabetes, %	12.6	20.7	5.4	** < 0.001**
CVD, %	8.7	11.9	5.9	
Triglycerides mg/dL	150 (148–153)	202 (198–206)	106 (104–107)	** < 0.001**
*HDL-C mg/dL*				
*Males*	46.6 (46.2–47.1)	42.2 (41.8–42.7)	51.7 (51.1–52.3)	** < 0.001**
*Females*	57.8 (57.3–58.3)	50.5 (50.0–51.0)	62.6 (62.0–63.3)	** < 0.001**
ALT (U/L)	23.9 (23.7–24.2)	28.2 (27.8–28.6)	20.2 (19.9–20.5)	** < 0.001**
AST (U/L)	24.0 (23.8–24.3)	25.4 (25.0–25.8)	22.8 (22.5–23.0)	** < 0.001**
*Smoking status*				
*Non-smokers*	59.5	55.5	63.9	** < 0.001**
*Previous smokers*	25.0	29.2	20.6	
*Smokers*	15.5	15.4	15.6	
Physical Activity (*z score*)	0.04 (0.02–0.05)	0.15 (0.12–0.18)	-0.08 **(**-0.10–-0.05)	** < 0.001**
*Nutrient Profile*				
*Energy Kcal*	1977 (1963–1990)	2014 (1995–2034)	1944 (1927–1961)	** < 0.001**
*Carbohydrates g/day*	240 (238–242)	242 (239–244)	240 (237–242)	0.172
*Protein g/day*	79.6 (78.9–80.4)	81.3 (80.3–82.4)	78.3 (77.3–79.2)	** < 0.001**
*Fats g/day*	77.9 (77.2–78.7)	81.0 (79.8–82.1)	75.4 (74.5–76.3)	** < 0.001**
*Saturated fat g/day*	25.3 (25.1–25.6)	26.5 (26.1–26.9)	24.4 (24.1–24.8)	** < 0.001**
*Monounsaturated fatty acids g/day*	27.5 (27.2–27.8)	28.5 (28.1–28.9)	26.7 (26.3–27.0)	** < 0.001**
*Polyunsaturated fatty acids g/day*	17.8 (17.6–18.1)	18.4 (18.1–18.8)	17.4 (17.1–17.7)	** < 0.001**
*Dietary fibre g/day*	16.9 (16.6–17.1)	16.5 (16.2–16.8)	17.2 (16.9–17.6)	** < 0.001**
*Diet Quality Indices*				
*HEI-2020 (out of 100)*	52.7 (52.3–53.2)	51.0 (50.7–51.3)	54.0 (53.6–54.4)	** < 0.001**
*MedDietScore (out of 55)*	22.2 (22.1–22.4)	21.5 (21.4–21.7)	22.9 (22.6–23.0)	** < 0.001**
*DASH score (out of 40)*	22.3 (22.1–22.5)	21.4 (21.3–21.6)	23.0 (22.8–23.2)	** < 0.001**

ALT, alanine aminotransferase; AST, aspartate aminotransferase; BMI, body mass index; DASH, dietary approach to stop hypertension; HDL-C, high-density lipoprotein cholesterol; HEI, healthy eating index; MedDietScore, Mediterranean diet score

MASLD was determined by the presence of hepatic steatosis estimated by fatty liver index ≥ 60, plus the presence of at least one of five cardiometabolic risk factors (CMRF) including: being overweight or obese, presence of diabetes, hypertension, hypertriglyceridemia, or low HDL levels

^*^Underweight (< 18.5 kg/m^2^) and normal weight (18.5–24.9 kg/m^2^) participants were combined due to the low prevalence of underweight (1.4%, n = 288) in the overall sample (n = 20,502) and its absence in the MASLD group, to enhance statistical power

^§^Weighted t-test and chi-square test used to determine associations between MASLD and non-MASLD groups for continuous and categorical variables, respectively

### Association of diet quality with MASLD and advanced fibrosis

For DQ scores, the association of continuous, per 1-SD change, and quartile- categorised scores with MASLD are reported in Table 2. The multivariate logistic regression analysis indicated that each unit increase in HEI-2020, MedDietScore and DASH score was associated with 2%–8% decrease odds for MASLD. Each 1-SD increase in HEI-2020, equivalent to a 12-point change, and MedDietScore, equivalent to a 4.8-point change, was associated with ~ 25% lower likelihood of MASLD, while a 1-SD increase in DASH, corresponding to a 4.7-point change, was associated with a 31% lower likelihood. Compared to the lowest DQ quartile, those in the highest quartile of HEI-2020 had significantly lower odds for MASLD (OR: 0.48, 95% CI 0.43–0.53); Similar association were observed for MedDietScore (OR: 0.49, 95% CI 0.45–0.55); and DASH score (OR: 0.40, 95% CI 0.36–0.45).

**Table 2:** Association of diet quality indices with MASLD and Advanced Fibrosis

Variables	HEI-2020	P Value	MedDietScore	*P* value	DASH score	*P* value
*Mean (95% CI) unless otherwise reported*						
*Association of diet quality indices with MASLD (n* = *20,502)*						
Continuous score	0.98 (0.97–0.98)	** < 0.001**	0.94 (0.93–0.95)	** < 0.001**	0.92 (0.91– 0.93)	** < 0.001**
1-SD increase*	0.75 (0.72–0.77)	** < 0.001**	0.75 (0.72–0.78)	** < 0.001**	0.69 (0.66–0.72)	** < 0.001**
Q1 (Lowest Quartile) **	1 (Reference)		1 (Reference)		1 (Reference)	
Q2	0.90 (0.81–1.01)	0.068	0.80 (0.72–0.89)	** < 0.001**	0.86 (0.78–0.95)	**0.004**
Q3	0.71 (0.64–0.80)	** < 0.001**	0.71 (0.63–0.79)	** < 0.001**	0.64 (0.57–0.73)	** < 0.001**
Q4	0.48 (0.43–0.53)	** < 0.001**	0.49 (0.45–0.55)	** < 0.001**	0.40 (0.36–0.45)	** < 0.001**
*Association of diet quality indices with advanced fibrosis within MASLD group (n* = *9,841)*						
Continuous score	0.98 (0.97–0.99)	**0.003**	0.96 (0.94–0.99)	**0.004**	0.95 (0.92–0.98)	** < 0.001**
1-SD increase*	0.83 (0.74–0.94)	**0.003**	0.84 (0.75–0.95)	**0.004**	0.78 (0.68–0.89)	** < 0.001**
Q1 (Lowest Quartile) **	1 (Reference)		1 (Reference)		1 (Reference)	
Q2	0.84 (0.63–1.14)	0.260	1.21 (0.94–1.57)	0.141	0.75 (0.52–1.08)	**0.117**
Q3	0.79 (0.56–1.10)	0.156	0.77 (0.58–1.03)	0.079	0.54 (0.39–0.75)	** < 0.001**
Q4	0.59 (0.44–0.79)	** < 0.001**	0.73 (0.54–0.98)	**0.036**	0.49 (0.34–0.72)	** < 0.001**

MASLD was determined by the presence of hepatic steatosis estimated by fatty liver index ≥ 60, plus the presence of at least one of five cardiometabolic risk factors (CMRF) including: being overweight or obese, presence of diabetes, hypertension, hypertriglyceridemia, or low HDL levels. Advanced fibrosis was determined by NAFLD fibrosis score of > 0.676

^§^Binary logistic regression analysis used to assess associations between MASLD and advanced fibrosis with diet quality scores. Analysis adjusted for age, sex, race, education, income to poverty ratio, smoking status, physical activity z score, energy intake, diabetes and CVD status

^*^Diet quality scores were standardised to a mean of 0 and a standard deviation (SD) of 1

The association between advanced fibrosis and DQ scores was assessed within the MASLD group (n = 9841) (Table 2)**.** The HEI-2020, MedDietScore and DASH score were associated with 2–5% significantly lowers odds of advanced fibrosis using continuous DQ scores. Furthermore, each 1-SD increase in HEI-2020, MedDietScore and DASH was significantly linked to 17%, 14% and 22% lower likelihood for advanced fibrosis. In the quartile-categorised scores, only the highest DQ quartile indicated significantly lower odds for advanced fibrosis: 0.49 (0.34–0.72) for DASH, 0.59 (0.44–0.79) for HEI-2020 and 0.73 (0.54–0.98) for MedDietScore, compared to the lowest quartile.

### Association of individual diet quality components with MASLD

**Fig. 2:**
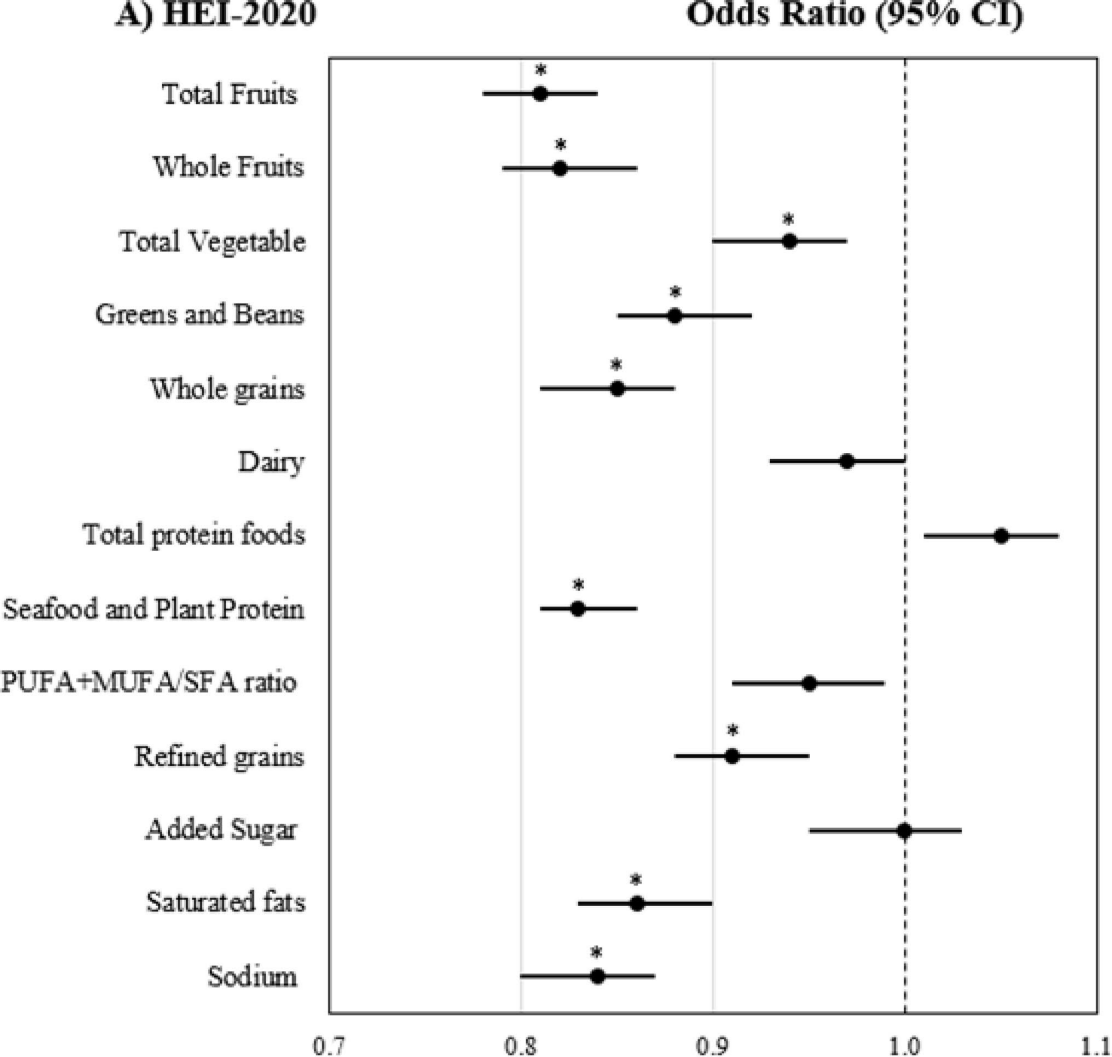

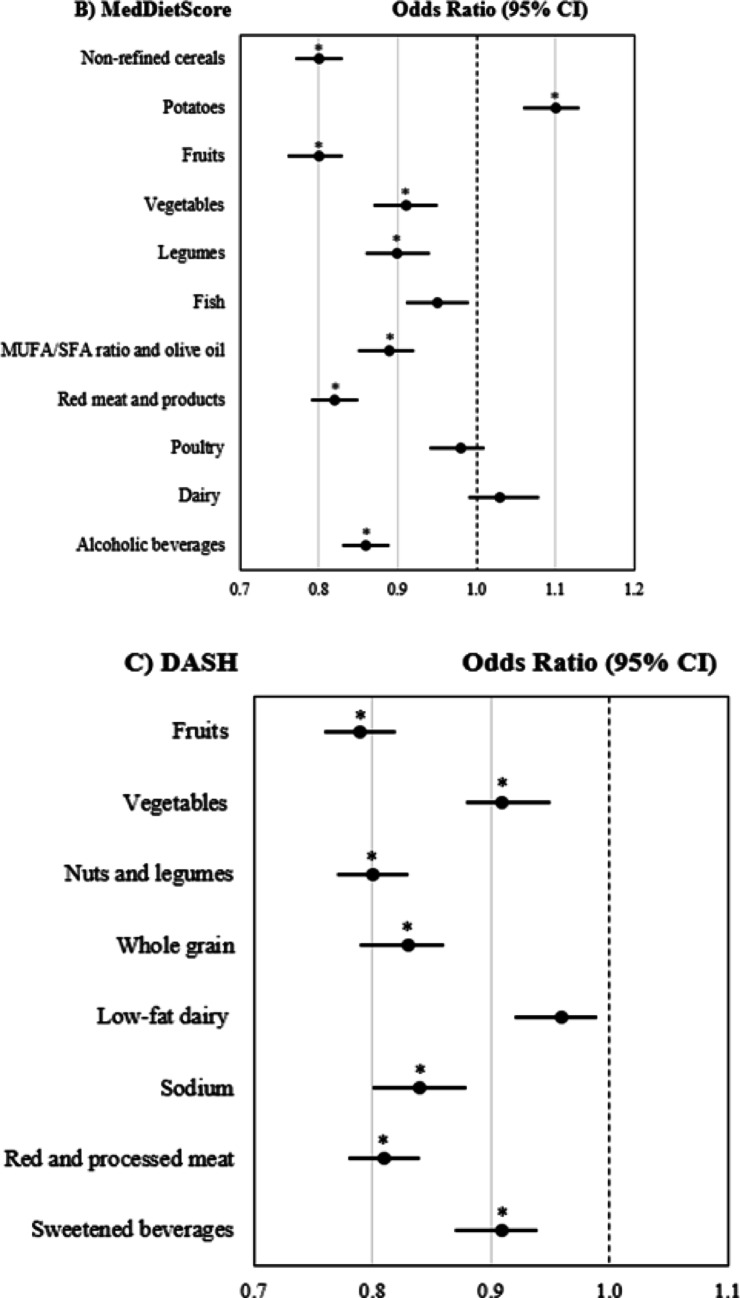
Forest plot of individual diet quality components score and MASLD risk. **A** HEI-2020 component scores **B** MedDietScore component scores **C** DASH component score. Scores standardised to a mean of 0 and standard deviation of 1. §Binary logistic regression analysis used to assess associations between MASLD and standardised diet quality scores. Analysis adjusted for age, sex, race, education, income to poverty ratio, smoking status, physical activity z score, energy intake, diabetes and CVD status. *Bonferroni* correction was applied to adjust for multiple comparisons across individual diet quality components. Statistical significance thresholds were set at *p* < 0.004 for HEI-2020, *p* < 0.005 for MedDietScore, and *p* < 0.006 for DASH*.* *Indicates statistical significance after Bonferroni correction

The standardised DQ components scores from HEI-2020, MedDietScore and DASH were assessed for their associations with MASLD (Fig. 2). The Bonferroni-adjusted analysis indicated that key adequacy components from HEI-2020, MedDietScore and DASH, such as whole grains, fruits, vegetables, legumes and seafood, were significantly associated with a lower likelihood of MASLD. Only the MUFA/SFA plus olive oil intake from MedDietScore was linked to lower odds of MASLD but not the PUFA + MUFA/SFA ratio from HEI-2020. The seafood and plant protein component from HEI-2020, but not the fish component from the MedDietScore, also showed statistical significance. The dairy component, regardless of being low- or full-fat, was not associated with MASLD. For moderation components, where higher scores reflect lower intakes, an inverse association with MASLD was observed particularly for refined grains, red and processed meats, saturated fats, and sodium (all *p*-values < 0.001). While lower added sugar intake was not significantly associated with MASLD (*p* = 0.825), sweetened beverage consumption showed a significant inverse association (*p* < 0.001).

## Longitudinal analysis

### Association of diet quality with all-cause mortality in the overall NHANES population

A total of 953 deaths occurred during a median follow-up of 9.3 years among 13,161 participants. The association of all-cause mortality with DQ scores are reported in Table 3 in a multivariable adjusted model. Each 1-SD increase in DQ was associated with significantly lower HR for mortality: HEI-2020, 0.93 (95% CI 0.86–0.99); MedDietScore, 0.92 (95% CI 0.86–0.99); DASH, 0.89 (95% CI 0.83–0.96). In the quartile-categorised analysis, only the MedDietScore was significantly associated with all-cause mortality. Compared to the lowest MedDietScore quartile, individuals in the highest quartile exhibited a significantly lower HR for all-cause mortality [0.78 (95% CI 0.65–0.95)] in the overall NHANES population.

**Table 3:** Association of diet quality indices with all-cause mortality (overall population)

Diet quality Indices	HEI-2020		MedDietScore		DASH diet	
	HR (95% CI)	*P* value	HR (95% CI)	*P* value	HR (95% CI)	*P* value
Continuous score	0.99 (0.98–0.99)	**0.045**	0.98 (0.96–0.99)	**0.035**	0.98 (0.96–0.99)	**0.003**
1-SD *	0.93 (0.86–0.99)	**0.045**	0.92 (0.86–0.99)	**0.035**	0.89 (0.83–0.96)	**0.003**
Q1 (Lowest Quartile) **	1 (Reference)		1 (Reference)		1 (Reference)	
Q2	0.95 (0.81–1.19)	0.655	0.78 (0.65–0.94)	**0.012**	1.06 (0.88–1.30)	0.505
Q3	0.87 (0.71–1.06)	0.144	0.83 (0.68–1.00)	0.051	0.95 (0.78–1.16)	0.602
Q4 (Highest Quartile)	0.82 (0.68–1.02)	0.060	0.78 (0.65–0.95)	**0.016**	0.85 (0.69–1.05)	0.142

^§^Multivariate adjusted Cox proportional hazard model used to determine associations between diet quality indices and all-cause mortality

Analysis adjusted for age, sex, race/ethnicity, education level, income to poverty ratio, diabetes, CVD status, smoking status, physical activity and mean energy intake

^**^Diet quality scores standardised to a mean of 0 and a standard deviation of 1

^**^Diet quality scores categorised into quartiles, with the lowest quartile (Q1) serving as the reference group

### Association of diet quality with all-cause mortality in those with and without MASLD

In the subgroup analysis, a significant protective association between DQ and all-cause mortality was observed in the non-MASLD population only. Using standardised scores, each 1-SD increase in DQ was associated with a lower HR for all-cause mortality: HEI-2020 (12-point increase), HR 0.87 (95% CI 0.79–0.97); MedDietScore (4.8-point increase), HR 0.89 (95% CI 0.80–0.98); and DASH (4.8-point increase), HR 0.82 (95% CI 0.74–0.91). In the quartile-based analysis, the highest DQ quartile for MedDietScore and DASH was associated with a 32% (*p* = 0.014) and 35% (*p* = 0.009) lower HR for all-cause mortality, respectively, compared to the lowest quartile (Table 4) in the non-MASLD population**.** However, none of the DQ score showed significant association with all-cause mortality in the MASLD population (Table 4).

**Table 4:** Association of diet quality with all-cause mortality in those with and without MASLD and advanced fibrosis

Diet quality Indices	HEI-2020		MedDietScore		DASH diet	
	HR (95% CI)	*P* value	HR (95% CI)	*P* value	HR (95% CI)	P value
*MASLD (n* = *6108)*						
Continuous score	0.99 (0.98–1.01)	0.657	0.99 (0.97–1.01)	0.451	0.99 (0.97–1.01)	0.487
1-SD *	0.98 (0.89–1.08)	0.657	0.96 (0.87–1.06)	0.451	0.96 (0.87–1.07)	0.487
Q1 (Lowest Quartile) **	1 (Reference)		1 (Reference)		1 (Reference)	
Q2	0.94 (0.73–1.22)	0.637	0.85 (0.65–1.09)	0.204	1.02 (0.77–1.34)	0.882
Q3	0.80 (0.62–1.05)	0.110	0.80 (0.61–1.04)	0.091	0.95 (0.74–1.23)	0.697
Q4 (Highest Quartile)	0.88 (0.68–1.13)	0.315	0.88 (0.68–1.14)	0.334	0.94 (0.72–1.23)	0.644
*Non-MASLD (n* = *7053)*						
Continuous score	0.99 (0.98–0.99)	**0.009**	0.97 (0.95–0.99)	**0.023**	0.96 (0.94–0.98)	** < 0.001**
1-SD *	0.87 (0.79–0.97)	**0.009**	0.89 (0.80–0.98)	**0.023**	0.82 (0.74–0.91)	** < 0.001**
Q1 (Lowest Quartile) **	1 (Reference)		1 (Reference)		1 (Reference)	
Q2	0.99 (0.74–1.32)	0.959	0.87 (0.65–1.15)	0.324	1.09 (0.82–1.46)	0.559
Q3	0.84 (0.62–1.12)	0.248	0.89 (0.67–1.18)	0.410	0.96 (0.71–1.29)	0.784
Q4 (Highest Quartile)	0.75 (0.56 -1.01)	0.055	0.68 (0.50–0.92)	**0.014**	0.65 (0.47–0.90)	**0.009**
*Advanced Fibrosis (n* = *517)*						
Continuous score	1.00 (0.98–1.01)	0.585	1.01 (0.96–1.05)	0.753	0.99 (0.94–1.03)	0.597
1-SD *	0.95 (0.77–1.16)	0.585	1.04 (0.83–1.29)	0.753	0.94 (0.76–1.17)	0.597
Q1 (Lowest Quartile) **	1 (Reference)		1 (Reference)		1 (Reference)	
Q2	1.34 (0.75–2.39)	0.321	1.02 (0.58–1.78)	0.951	0.76 (0.43–1.38)	0.378
Q3	1.01 (0.57–1.80)	0.971	1.04 (0.58–1.85)	0.907	0.72 (0.41–1.27)	0.256
Q4 (Highest Quartile)	0.98 (0.55–1.74)	0.941	1.05 (0.59–1.86)	0.880	0.74 (0.42–1.34)	0.325
*Without Advanced Fibrosis (n* = *5552)*						
Continuous score	1.00 (0.99–1.01)	0.874	0.99 (0.96–1.01)	0.274	0.99 (0.97–1.02)	0.647
1-SD *	0.99 (0.89–1.11)	0.874	0.94 (0.83–1.05)	0.274	0.97 (0.87–1.09)	0.647
Q1 (Lowest Quartile) **	1 (Reference)		1 (Reference)		1 (Reference)	
Q2	0.88 (0.65–1.18)	0.395	0.83 (0.61–1.11)	0.205	1.13 (0.83–1.55)	0.432
Q3	0.78 (0.57–1.07)	0.120	0.74 (0.55–1.01)	0.056	1.03 (0.76–1.36)	0.903
Q4 (Highest Quartile)	0.87 (0.64–1.17)	0.358	0.84 (0.63–1.13)	0.247	1.01 (0.74–1.37)	0.936

^§^Multivariate adjusted Cox proportional hazard model used to determine associations between diet quality indices and all-cause mortality

Analysis adjusted for age, sex, race/ethnicity, education level, income to poverty ratio, diabetes, CVD status, smoking status, physical activity and mean energy intake

^*^Diet quality scores standardised to a mean of 0 and a standard deviation of 1

^**^Diet quality scores categorised into quartiles, with the lowest quartile (Q1) serving as the reference group

### Association of diet quality with all-cause mortality in those with and without advanced fibrosis in the MASLD group

None of the DQ scores were associated with all-cause mortality among individuals with or without advanced fibrosis in the MASLD group, regardless of whether the scores were treated as continuous, standardised, or categorised into quartiles (Table 4).

## Discussion

This study, which includes both cross-sectional and longitudinal analyses of DQ and their associations with MASLD and mortality in a large, nationally representative NHANES database (2005–2020), showed that increased adherence to the HEI-2020, MedDietScore, and DASH dietary patterns were significantly associated with a lower likelihood of MASLD and advanced fibrosis. Furthermore, longitudinal analysis showed that higher HEI-2020, MedDietScore, and DASH scores were protective against all-cause mortality in the overall population and among individuals without MASLD. However, no significant association between DQ and all-cause mortality was observed among individuals with MASLD regardless of advanced fibrosis status. This suggests that while DQ plays a preventive role in the development of MASLD and contributes to overall longevity, its impact on mortality may be diminished once the disease is established, possibly due to underlying metabolic complications or disease severity. Diet may act as an indirect contributor, whereas metabolic complications, often secondary to an unhealthy lifestyle, serve as an independent contributing factor for mortality in the MASLD population [52, 53]. Our findings also suggest that early dietary intervention, particularly in people at-risk of MASLD, such as those with excess weight, insulin resistance, or metabolic syndrome, should be considered a priori as a prevention strategy.

Although there is evidence supporting the role of excess energy intake in the development of MASLD [54], the current study demonstrates that the relationship between diet and MASLD extends beyond energy excess. The analysis showed that all DQ scores, whether treated as continuous or categorised into quartiles, were associated with a lower likelihood of MASLD and advanced fibrosis independent of energy intake. These findings also support the adoption of dietary patterns such as the HEI, Med diet, or DASH to reduce the risk of developing MASLD, when compared to more restrictive eating behaviours like low-fat or very low-carbohydrate diets, which can be challenging to maintain and lack long-term safety evidence [32]. There is also evidence that both the Med diet and DASH diet are effective for weight loss in overweight and obese adults with metabolic syndrome [55, 56]. Importantly, these dietary patterns can be modified to accommodate individual needs and preferences, seasonal food availability, and affordability, thereby enhancing their adoptability across multiethnic populations [33].

In line with previous evidence [9], our analysis showed that higher scores on key adequacy components across all DQ indices, namely whole grains, fruits, vegetables, plant and seafood protein, and the MUFA/SFA ratio, were associated with a lower likelihood of MASLD. A possible mechanism underlying these associations may involve the anti-steatogenic and hypolipidemic effects of these components, including dietary fibre, which is fermented into short-chain fatty acids that regulate hepatic fat accumulation and inflammation via the gut–liver axis [57]. Phenolic compounds in fruits, vegetables, and legumes promote hepatic lipolysis, inhibit lipogenesis, and counteract oxidative stress and fibrogenesis [58]. Omega-3 PUFAs found in seafood inhibit the synthesis of total cholesterol and triglycerides, suppress de novo lipogenesis, enhance lipid oxidation, and exhibit anti-inflammatory and anti-fibrotic activities in the hepatocytes [59]. Extra virgin olive oil, a key component of the Med diet enhances fatty acid oxidation, inhibits lipogenesis, improves insulin sensitivity, and attenuates hepatic steatosis owing to its high MUFA content and antioxidant capacity [60]. Its cardiometabolic benefits are further enhanced by the use of high-quality oils, particularly those rich in polyphenols [61]. For the moderating components, lower intakes of refined grains, red and processed meats, sodium, sugar-sweetened beverages and moderate alcohol consumption have been associated with a lower likelihood of MASLD in this study. These components, characteristic of a typical Western-style diet, have been significantly associated with MASLD-related liver mortality in a recent Global Burden of Disease (2022) study [62].

Another key finding from our study is that all DQ indices were inversely associated with all-cause mortality in the overall population and those without MASLD, but not in individuals with MASLD, regardless of fibrosis status. There is inconsistency in findings in relations to the association of DQ with all-cause mortality in people with established MASLD. Yoo et al. (2020), using data from the third NHANES survey, reported that higher DQ, assessed using the HEI-1995, was inversely associated with ultrasonographic diagnosed MASLD [63]. A significant protective association with all-cause, CVD, and cancer-related mortality was observed in the overall population and among individuals without NAFLD; however, no such association was observed in the NAFLD group [63]. In contrast, another study using data from the third NHANES survey, which applied the metabolic dysfunction-associated fatty liver disease (MAFLD) definition, reported a significant protective association between DQ assessed using the HEI-2005 and all-cause mortality in the MAFLD population [64]. The study also found a significant association between DQ and mortality among individuals who were less physically active, but not among those who were physically active, based on the median physical activity score [64]. A recent study using data from NHANES 2005–2018, comprising a population with MASLD (n = 3634), assessed the association between DQ and all-cause mortality [65]. DQ was assessed using the HEI-2020, DASH, alternate Med diet, alternate Healthy Eating Index, and the Dietary Inflammatory Index, all of which showed significant protective associations with all-cause mortality [65]. Unlike the latter two studies, the current study and that by Yoo et al. (2020) adjusted the analysis for physical activity, smoking status, total energy and alcohol intake which may help elucidate the influence of modifiable risk factors on mortality.

Our findings also suggest the timing of improvements in DQ, particularly before the onset or progression of disease, is important in preventing adverse health outcomes, as reported by previous studies [66, 67]. In the NHANES, the association between HEI-2015 and all-cause mortality was absent among individuals with obesity and severe inflammation (CRP > 3 mg/L), but remained significant in those with moderate (CRP 1–3 mg/L) or low inflammatory burden (CRP < 1 mg/L) [66]. Findings from the Dutch Lifelines cohort (n = 40,892) also reported no association between DQ and mortality among adults aged ≥ 50 years with one or more cardiometabolic diseases; however, significant associations were observed in individuals without any CVD and those with only T2DM [67]. These findings underscore the potential importance of early MASLD prevention, particularly among individuals with excess weight, insulin resistance, or metabolic syndrome. Moreover, the null associations may reflect the unique nutritional requirements of those with multiple metabolic comorbidities, which are not adequately captured by standard DQ indices developed for the general population. Lastly, post-diagnosis changes in dietary behaviours may also attenuate the observed associations between DQ and mortality.

Another notable finding from this study is the overall low adherence to the assessed dietary patterns. Among participants in the highest quartile, mean DQ scores were 68.6 out of 100 for HEI-2020, 28.6 out of 55 for the MedDietScore, and 28.8 out of 40 for the DASH score. These values indicate that even participants with relatively higher adherence did not reach the thresholds typically considered indicative of high DQ, particularly an HEI score of ≥ 80 in the U.S. population [68]. While defined cut-offs for high adherence to the MedDietScore and DASH are lacking, the highest quartile means in NHANES were lower than those reported in other Western populations: MedDietScore (28.6 *vs* 36) in a Med population [69] and DASH score (28.8 *vs* 34.21) in the European general population aged 50–69 years [70]. This is further reflected in the clinical characteristics of this NHANES population, with a mean BMI of 29.3 kg/m^2^ and nearly half of the population identified as having MASLD (47.8%). Our study highlights that the DQ amongst the U.S. general population is relatively poor, but modest improvements may play a key role to reduce the growing MASLD burden.

This study has several strengths. First, it utilised the large, nationally representative NHANES database (2005–2020) with a substantial sample size, incorporating both cross-sectional and longitudinal analyses. Second, the availability of extensive sociodemographic, clinical, and lifestyle covariates enabled us to account for various factors that may confound the associations between DQ, MASLD and all-cause mortality, thereby enhancing the robustness of our analysis. Third, the initial five years of follow-up were excluded from the longitudinal analysis to minimise the risk of reverse causality. Finally, subgroup analyses were conducted for individuals with and without MASLD, as well as those with and without advanced fibrosis, using group-specific DQ quartile cut-offs to understand the impact of adherence levels to dietary patterns on outcomes. There were several limitations to this study. First, MASLD and advanced fibrosis were defined using the FLI and NFS, respectively rather than gold standard biopsy or imaging which are more accurate but often not practical to obtain in large national datasets. Nonetheless, both indices showed acceptable diagnostic performance in NHANES 2017–2020, with FLI showing 79.6% sensitivity for predicting MASLD and NFS showing 92.5% specificity for predicting advanced fibrosis [71]. Second, dietary intake in NHANES was assessed via 24-h recalls, which have been susceptible to various sources of bias and measurement error [37, 72]. These include recall bias, social desirability bias in reporting foods perceived as healthy or unhealthy, and inaccuracies in portion size estimation [37]. To mitigate recall bias, NHANES has employed the Automated Multiple-Pass Method to capture accurate food recalls and reduce respondent burden. This study has also excluded individuals with implausible energy intakes and only those with two reliable 24 h dietary recalls available were included. Although NHANES has used 2 × 24 h dietary recall to capture day-to-day variation, this approach may not accurately capture habitual long term dietary pattern critical for MASLD onset and progression. Third, the frequency of olive consumption, a component of the MedDietScore, was not available in NHANES; therefore, this component was substituted with the ratio of MUFA: SFA, combined with olive oil intake expressed in grams. Fourth, we were unable to assess cause-specific mortality, such as CVD or cancer-related deaths, due to the low number of events (< 10%) in our dataset, limiting the analysis to all-cause mortality. Finally, data on liver-specific mortality were not available in the NHANES public-use linked mortality files, which may be critical for understanding the association between DQ and liver-related mortality in people with MASLD.

Our findings support the adoption of healthy dietary patterns in line with a high HEI, the Med diet, and/or the DASH diet for the prevention and management of MASLD. Clinical practice guidelines for MASLD currently emphasise the Med diet, which is supported by these findings, which also reiterate the literature to suggest that improvements in DQ overall confer benefits [22]. When culturally and personally tailored, such patterns may enhance long-term adherence and effectiveness in preventing MASLD and mitigating associated adverse health outcomes.

## Conclusion

This study found that better DQ, as assessed by the HEI-2020, MedDietScore, and DASH score, was associated with a lower likelihood of MASLD and advanced fibrosis among individuals with established MASLD. A higher-quality diet was also linked to a reduced risk of all-cause mortality in the overall population and in those without MASLD, but this protective association was not observed in individuals with MASLD, regardless of fibrosis status. These findings suggest that the timing of prevention strategies (e.g., prior to the establishment of disease) may be a key criterion when making dietary improvements to reduce mortality risk.

## Supplementary Information

Below is the link to the electronic supplementary material.

Supplementary Material 1

## Data Availability

The data for this study are available from the corresponding author, Elena S. George, upon request.
