# Supplementary material for: Higher diet quality is associated with a lower prevalence of MASLD and adverse health outcomes: insights from NHANES 2005 to 2020
Source: Eur J Nutr. 2025 Oct 6;64(7):289. doi: 10.1007/s00394-025-03809-4 (PMC12500818; doi:10.1007/s00394-025-03809-4)
Supplement: Supplementary file 1 — Supplementary Material 1 [file 394_2025_3809_MOESM1_ESM.docx]

**Supplementary File S1**

**Table S1 Components of Health Eating Index-2020**

| **Component** | **Maximum Points** | **Standard for Maximum Score** | **Standard for Minimum Score of Zero** |
| --- | --- | --- | --- |
| **Adequacy Components** |  |  |  |
| Total Fruits | 5 | ≥0.8 cup equiv. per 1,000 kcal | No Fruits |
| Whole Fruits | 5 | ≥0.4 cup equiv. per 1,000 kcal | No Whole Fruits |
| Total Vegetables | 5 | ≥1.1 cup equiv. per 1,000 kcal | No Vegetables |
| Greens and Beans | 5 | ≥0.2 cup equiv. per 1,000 kcal | No Dark Green Vegetables or Legumes |
| Whole Grains | 10 | ≥1.5 oz equiv. per 1,000 kcal | No Whole Grains |
| Dairy | 10 | ≥1.3 cup equiv. per 1,000 kcal | No Dairy |
| Total Protein Foods | 5 | ≥2.5 oz equiv. per 1,000 kcal | No Protein Foods |
| Seafood and Plant Proteins | 5 | ≥ 0.8 oz equiv. per 1,000 kcal | No Seafood or Plant Proteins |
| Fatty Acids | 10 | (PUFAs + MUFAs)/SFAs ≥2.5 | (PUFAs + MUFAs)/SFAs ≤1.2 |
| **Moderation Components** |  |  |  |
| Refined Grains | 10 | ≤1.8 oz equiv. per 1,000 kcal | ≥4.3 oz equiv. per 1,000 kcal |
| Added Sugars | 10 | ≤6.5% of energy | ≥26% of energy |
| Sodium | 10 | ≤1.1 gram per 1,000 kcal | ≥2.0 grams per 1,000 kcal |
| Saturated Fats | 10 | ≤8% of energy | ≥16% of energy |
| **Abbreviations** cup equiv., cup equivalents; kcal, kilocalories; oz. equiv.=ounce equivalents; g, grams; mg, milligrams | | | |

**Table S2 Components of Mediterranean Diet Score (MedDietScore)**

| **Components** | **Frequency of consumption**  **(Servings per week or otherwise stated)** | | | | | |
| --- | --- | --- | --- | --- | --- | --- |
| Non-refined cereals | Never | 1–6 | 7–12 | 13–18 | 19–31 | >32 |
| Potatoes | Never | 1–4 | 5–8 | 9–12 | 13–18 | >18 |
| Fruits | Never | 1–4 | 5–8 | 9–15 | 16–21 | >22 |
| Vegetables | Never | 1–6 | 7–12 | 13–20 | 21–32 | >33 |
| Legumes | Never | <1 | 1–2 | 3–4 | 5–6 | >6 |
| Fish | Never | <1 | 1–2 | 3–4 | 5–6 | >6 |
| MUFA/SFA ratio | >0.4 | 0.4 – 0.8 | >0.8 – 1.2 | >1.2 – 1.6 | >1.6 – 2 | >2 |
| ***Scoring Criteria*** | **0** | **1** | **2** | **3** | **4** | **5** |
|  |  |  |  |  |  |  |
| Red meat and products | ≤1 | 2–3 | 4–5 | 6–7 | 8–10 | >10 |
| Poultry | ≤3 | 4–5 | 5–6 | 7–8 | 9–10 | >10 |
| Full fat dairy products | ≤10 | 11–15 | 16–20 | 21–28 | 29–30 | >30 |
| Alcoholic beverages ml/day | <300 | 300 | 400 | 500 | 600 | >700 or 0 |
| ***Scoring Criteria (Reverse)*** | **5** | **4** | **3** | **2** | **1** | **0** |
| **Abbreviations** ml, millilitre  **Note** The scoring criterion for the MUFA/SFA ratio is out of 2.5 points instead of the usual 5. An additional 2.5 points are allocated for the inclusion of any amount of olive oil in the diet. For example, if a participant has a MUFA/SFA ratio greater than 2, they are awarded 2.5 points, and an additional 2.5 points if olive oil is also consumed. | | | | | | |

**Table S3 Components of Dietary Approach to Stop Hypertension Score**

| **Components** | **Foods** | **Scoring Criteria** |
| --- | --- | --- |
|  |  |  |
| Fruits | All fruits and fruit juices | 5-1 |
| Vegetables | All vegetables except potatoes and legumes | 5-1 |
| Nuts and legumes | Nuts and peanut butter, dried beans, peas, tofu | 5-1 |
| Whole grains | Brown rice, dark breads, cooked cereal, whole grain cereal, other grains, popcorns, wheat germ, bran | 5-1 |
| Low-fat dairy | Skim milk, yogurt, cottage cheese | 5-1 |
| ***Scoring Criteria*** | ***5 points for maximum intake (Q5) and 1 point for minimum intake (Q1)*** | |
|  |  |  |
| Sodium | Sum of sodium content in all foods | 1-5 |
| Red and processed meats | Beef, pork, lamb, deli meats, organ meats, hot dogs, bacon | 1-5 |
| Sweetened beverages | Carbonated and noncarbonated sweetened beverages | 1-5 |
| ***Scoring Criteria (Reverse)*** | ***5 points for minimum intake (Q1) and 1 point for maximum intake (Q5)*** | |
| **Abbreviations** Q, quintiles. | | |
